# An Enhanced Bimetallic Optical Fiber SPR Biosensor Using Graphene Oxide for the Label-Free and Sensitive Detection of Human IgG

**DOI:** 10.3390/s25051630

**Published:** 2025-03-06

**Authors:** Qiang Xu, Huiting Yin, Mei Cui, Renliang Huang, Rongxin Su

**Affiliations:** 1 Tianjin Key Laboratory for Marine Environmental Research and Service, School of Marine Science and Technology, Tianjin University, Tianjin 300072, China; xuqiang0078@163.com (Q.X.); tjuhrl@tju.edu.cn (R.H.); 2 Ningbo Key Laboratory of Green Petrochemical Carbon Emission Reduction Technology and Equipment, Zhejiang Institute of Tianjin University, Ningbo 315201, China; 3 State Key Laboratory of Chemical Engineering, Tianjin Key Laboratory of Membrane Science and Desalination Technology, School of Chemical Engineering and Technology, Tianjin University, Tianjin 300072, China; meicui@tju.edu.cn

**Keywords:** bimetal sensor, fiber-optic SPR, graphene oxide, antibody, human IgG

## Abstract

A fiber-reinforced SPR sensor based on silver-nucleated gold-shell bimetallic nanoparticles and graphene oxide was developed and applied to human IgG detection. The refractive index (RI) sensitivity of the Ag@Au/GO fiber SPR sensor is as high as 4715.9 nm/RIU in the RI range of 1.333–1.365. Staphylococcus aureus protein A (SPA) can specifically recognize and bind to the fragment crystallizable (Fc) of the antibody; it facilitates the highly targeted immobilization of the antibody. SPA and rabbit anti-human IgG were immobilized on the surface of the Ag@Au/GO fiber SPR sensor for the detection of different concentrations of human IgG with a sensitivity of 0.53 nm/μg/mL and detection limits of 0.037 μg/mL. This biosensor based on the mixed structure of GO and Ag@Au combined the common advantages of the two materials. Therefore, our study provides a simple platform for biological analysis and has a good application prospect.

## 1. Introduction

Immunoglobulin G (IgG) is the most important class of antibodies in the body’s immune system [1]. Systemic lupus erythematosus, hyperthyroidism, and other autoimmune diseases are caused by abnormal IgG concentrations; they can lead to specific infectious diseases in severe cases, so their concentration detection is crucial in the fields of medical diagnosis, drug research, and quality monitoring [2]. At present, commonly used methods for the detection of human IgG include enzyme-linked immunosorbent assay (ELISA) [3,4], fluorescence immunoassay [5], electrochemical sensor [6,7], etc. ELISA requires high enzyme activity, and the changes in ambient temperature may affect enzyme activity. Fluorescence immunoassays are time-consuming. Electrochemical sensors have a limited concentration measurement range with low sensitivity and poor stability. Therefore, there is an urgent need to develop a high-precision analytical detection method. Traditional SPR sensors have a limited ability to detect low-molecular-weight biomolecules and biomolecules at ng/mL concentration levels. In order to improve the detection sensitivity of SPR sensors to small changes in the refractive index at the interface between metal films and analytes, new nanomaterials have been introduced to further improve the detection ability of optical fiber SPR sensors, and the analytical performance has been strengthened by fabricating new sensing structures [8,9,10]. Compared with gold nanoparticles, silver ones are more sensitive to changes in the thickness and refractive index of the sensor, and the good optical properties of silver nanoparticles will greatly promote SPR sensing under the condition of effectively preventing oxidation. Wang et al. [11] improved the antioxidant capacity of silver films by modifying GO on the surface of silver film sensors, preventing oxygen molecules from passing through the surface of the silver film, increasing the refractive index sensitivity to 3311 nm/RIU, and fixing the surface of the sensor with SPA to functionalize it; the sensitivity of immunodetection of human IgG was as high as 0.4985 nm/µg/mL.

Two-dimensional nanomaterial metals include metal–organic frameworks, graphite oxide, carboxylated graphene oxide, etc. Zhu et al. [12] proposed an enhanced SPR optical fiber biosensor using Ti_3_C_2_T_x_ MXene/AuNPs for the label-free and sensitive detection of human IgG, fabricated using a heterostructure optical fiber coated with Au film/AuNPs and Ti_3_C_2_T_x_ MXene biofunctionalized with goat anti-human IgG by polydopamine (PDA). However, its refractive index sensitivity was low, at 2804.5 nm/RIU, and the detection sensitivity of human IgG was 1.7046 nm/µg/mL. Graphene materials have a large specific surface area, good electrical conductivity, ultra-high mechanical strength, and biocompatibility. Graphene and graphene oxide (GO) not only have an incremental effect on biomolecule affinity, but also improve anti-pollution and antioxidant capacity [13,14]. GO and its derivatives have been widely used in many fields such as transparent electrodes [15], energy storage [16], biosensors [17], drug delivery [18], and catalysis [19]. Zhao et al. [20] proposed an SPR fiber sensor established on gold nanoparticle/GO heterostructures and Au film coupling enhancement, and the RI sensitivity was up to 3436.2 nm/RIU. In recent years, GO has been widely used in the manufacture of immunosensors or other biosensors due to its simple surface modification and high charge mobility, and the carboxyl groups on the surface of GO make it easy to further functionalize different kinds of biomolecules. Graphene can also be used as a surface modifier for fiber optic sensors, providing a protective barrier for biologics in real-time spectroscopic experiments.

At present, the surface plasmon resonance sensor has become a breakthrough biosensing technology that solves many of the limitations of traditional technologies and has obvious advantages. In particular, it can detect biomolecular interactions in real time and without labeling. Unlike traditional methods such as enzyme-linked immunosorbent testing, which often involve complex labeling and multi-step detection, SPR can directly and in real time measure target interactions on the sensor surface. In recent years, plasma biosensors have made significant progress, especially in improving sensitivity, selectivity, and miniaturization. These developments have transformed it from a research-based technology to a versatile detection tool. One of the most important breakthroughs is the integration of nanomaterials, which greatly improves the performance of SPR sensors by amplifying plasma signals and improving the overall sensitivity of detection [21].

**Figure 1:**
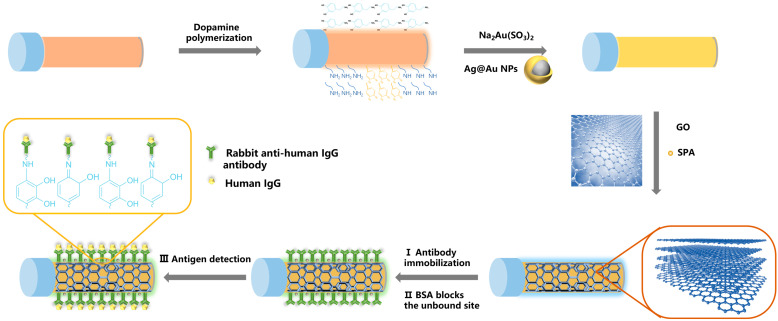
Schematic diagram of the preparation of graphene oxide-enhanced silver-nucleated gold-shell bimetallic fiber SPR sensor and antigen–antibody immunodetection.

The sensor in our study can create an enhanced surface plasmon resonance effect through gold and silver bimetallic nanoparticles and graphene oxide modification, which can amplify the sensing signal and greatly improve the sensitivity of detection. Graphene oxide can achieve an immunoassay effect by adsorbing specific antibodies that recognize analytes, and the overall performance is better than that of sensors using only a single gold film or graphene oxide [22]. In this study, the refractive index detection sensitivity is as high as 4715.9 nm/RIU and the sensitivity of the antigen–antibody specific binding test for human IgG was up to 0.53 nm/μg/mL. This biosensor based on the mixed structure of graphene oxide and gold–silver combined the common advantages of the two materials and has a good application prospect. It is of great significance to develop an efficient and convenient human IgG detection method that can be monitored in real time. In this experiment, a bimetallic graphene oxide-enhanced Ag@Au/GO fiber optical SPR sensor with silver-core gold-shell nanoparticles was used to highly immobilize antibodies by SPA and then used to detect different concentrations of human IgG (As shown in Figure 1).

## 2. Materials and Methods

### 2.1. Materials

Dopamine hydrochloride (DA), Bovine Serum Albumin (BSA), graphene oxide, staphylococcal protein A (SPA), ethanolamine, and sodium hydroxide (NaOH) were purchased from Shanghai Aladdin Biochemical Science and Technology Co., Ltd. (Shanghai, China). Rabbit anti-human IgG antibody, human IgG, and goat IgG were purchased from Shanghai Zhuoshi Biotechnology Co (Shanghai, China). The rabbit anti-human IgG antibody, human IgG, and goat IgG were stored at −20 °C, while all other biological reagents were stored at 4 °C. Sodium phosphate-buffered saline was used as the buffer. All the solutions were prepared in deionized water (DI water, specific resistivity >18 MΩ) at 25 °C.

### 2.2. Preparation of Ag@Au/GO Fiber Optic SPR Sensors

Based on our previous study [23], a new fiber SPR sensor (Au-Ag@Au) was fabricated by the in situ growth of gold and silver nanoparticles on the surface of optical fibers using the polydopamine adhesion and displacement reaction strategy, and by growing gold films via wet electroless plating (ELP) and sulfite complexation. The sensor was immersed in solutions with different refractive indices to obtain unmodified GO with a sensitivity of 2683.9 nm/RIU (the refractive index range is 1.333–1.365). The Ag@Au bimetallic fiber optic SPR sensor was prepared by polydopamine (PDA) adhesion and sodium sulfite complexation, and then the sensing area was immersed in DA solution (2 mg/mL, 30 min) to generate amino groups on the surface of the gold film, which were used to modify GO, SPA, and GO-SPA, respectively, in the subsequent process. The sensing area of the Ag@Au fiber SPR sensor was immersed in different concentrations of GO solution (0.5 mg/mL, 1 mg/mL, 1.5 mg/mL, 2 mg/mL), the spectral changes in the sensor were recorded, and the changes in sensor sensitivity at different concentrations were compared to obtain the optimal modified GO concentration. Second, the sensing area of the Ag@Au-fiber SPR sensor modified with PDA was immersed in SPA solution at different concentrations (25 μg/mL, 50 μg/mL, 75 μg/mL, 100 μg/mL) separately, and the spectral changes in the sensor at different concentrations were compared. Finally, the Ag@Au fiber SPR sensor was modified with GO and SPA at the same time, the Ag@Au/GO fiber SPR sensor was immersed in an EDC/NHS mixed solution (0.4 mol/L EDC: 0.1 mol/L NHS = 1:1 *v*/*v*) for 20 min at room temperature, and the sensor surface was washed with deionized water and dried with nitrogen. At this time, the carboxyl group of GO on the sensor surface was activated and could be combined with the compound containing amino groups. The sensor was then immersed in SPA solution to increase the amount of antibody fixation.

### 2.3. Ag@Au/GO Sensor Antibody Fixation and Antigen–Antibody Immunoassay

The silver-core gold-shell fiber SPR sensors modified with GO and SPA and GO-SPA were immersed in 100 µg/mL human IgG solution for 2 h, respectively, and the offsets of the reflectance spectrum and resonance wavelength were obtained. In order to achieve modification on the surface of the fiber, the unbound rabbit anti-human IgG was rinsed with PBS solution, and finally, the binding site on the surface of the fiber was closed with ethanolamine solution to block the unbound carboxyl group. The optical fiber SPR sensor has the advantages of the continuous and real-time monitoring of signal response, and the optical fiber SPR system is used to monitor the resonance wavelength shift caused by the interaction between antibody and antigen.

The Ag@Au/GO sensor with immobilized rabbit anti-human antibody was used to detect the human IgG antigen; its sensing area was immersed in different concentrations of human IgG antigen solution (10 μg/mL, 20 μg/mL, 40 μg/mL, 60 μg/mL, 80 μg/mL, 100 μg/mL) for 30 min. The measured reflectance spectra were saved, and the sensor was thoroughly rinsed with buffer to remove unbound antigen. The detected sensor was immersed in 10 mM NaOH solution to eliminate the interaction between antigen and antibody, so that the sensor with immobilized antibodies could be regenerated and reused for detection. In the process of the antigen–antibody immunoassay test, we carried out the dissociation between antigen and antibody on the SPR sensor adsorbed with antigen to ensure the normal progress of the experiment. At the end of the detection of different IgG concentrations, the sensor was immersed in 10 mM NaOH solution for dissociation and rinsed with PBS buffer to destroy the binding between human IgG and rabbit anti-human IgG [11,24,25]. It is important to note that the concentrations of SPA and rabbit anti-human IgG are very large compared to that of human IgG, so binding the human IgG to the biomodified layer with respect to the amount of human IgG measured can be considered sufficient.

### 2.4. Evaluation of Specificity and Selectivity of Antigen–Antibody Immunoassays and Sensor Stability Testing

In order to evaluate the specificity and selectivity of the assay, we used the same method as that of human IgG detection to detect multicomponent solutions. The Ag@Au/GO sensor immobilized with antibodies was used to detect a certain concentration of human IgG, BSA, goat IgG, BSA + human IgG, and goat IgG + human IgG, and the test results were compared with the results of human IgG detection. In order to determine the detection stability of the sensor, the sensor with fixed antibodies was stored in a −4 °C freezer for 0 days, 5 days, 10 days, and 15 days.

## 3. Results and Discussion

### 3.1. Characterization of Fiber Optic SPR Sensors for GO Functionalization

**Figure 2:**
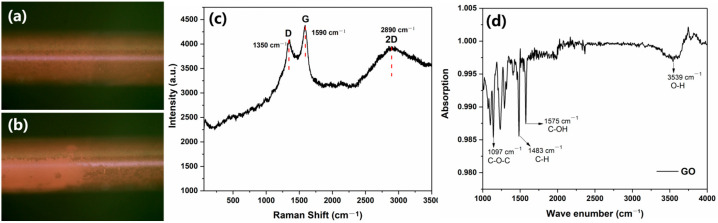
Optical image of GO-coated fiber micrographs of (**a**) uncoated and (**b**) GO-coated fibers. Raman spectra (**c**) and FT-IR spectrum (**d**) of GO coated on the surface of the SPR sensor.

The surface of the GO-modified fiber SPR sensor was characterized. Figure 2a,b showed the comparison of the optical images of GO coated onto the surface of a fiber optic sensor under a polarizing microscope. Figure 2a shows a polarizing microscope image of the optical fiber surface without GO, and Figure 2b shows a polarizing microscope image of the optical fiber surface measured after GO coating. Compared with Figure 2a, Figure 2b shows that GO is thoroughly spread on the optical fiber surface after coating. Compared to the uncoated images (Figure 2a), Figure 2b shows that the GO layer was clearly retouched to the sensor surface, which is consistent with the results in other studies [26]. Raman spectroscopy is an analytical tool used to characterize carbon-based materials. Figure 2c shows the Raman spectrum of the GO film on the sensor surface, which consisted of three main peaks: D (1350 cm^−1^), G (1590 cm^−1^), and 2D (2890 cm^−1^). The D peak was due to defects caused by the adhesion of hydroxyl and epoxy groups on the carbon-based plane and edges, and the G peak was due to first-order scattering in E_2g_ mode. The 2D peak was shown at 2890 cm^−1^, where it proved that the graphene had a monolayer structure [27]. The FT-IR spectrum of GO shown in Figure 2d contained the peaks of asymmetric C-O-C stretching vibrations (1097 cm^−1^), saturated C-H bending vibrations (1483 cm^−1^), and O-H stretching vibrations (3539 cm^−1^), and the peak at 1575 cm^−1^ was formed during the tensile shaking of the carboxyl group at the edge of the conjugated carbonyl group (-C=O-).

**Figure 3:**
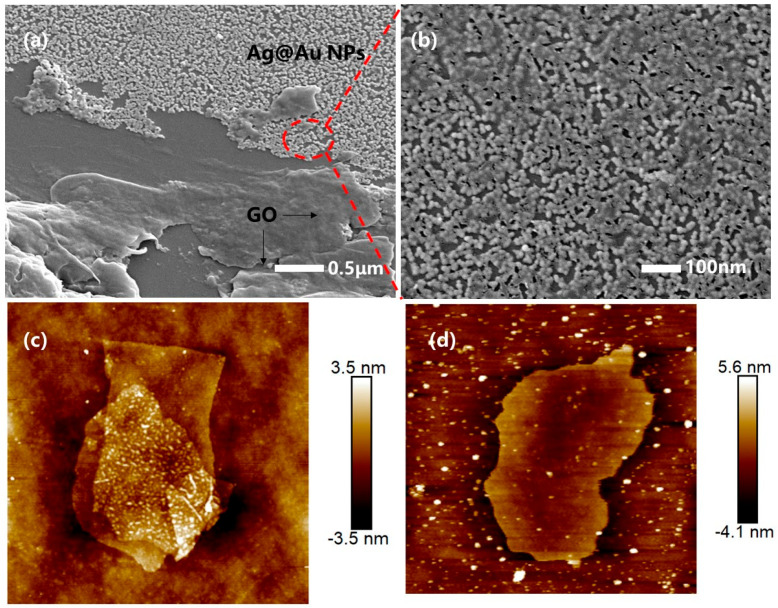
SEM images (**a**,**b**) and AFM images (**c**,**d**) of the GO used to modify the fiber optic SPR biosensor. (The red dotted line area in (**a**) is enlarged and shown as (**b**)).

As shown in Figure 3, SEM was used to characterize the surface of the silver-core-gold-shell bimetal and graphene oxide-reinforced fiber SPR sensors. GO was successfully grown on the surface of the Ag@Au film sensor, it was in a lamellar structure on the surface of the gold–silver nanoparticles, and was in a folded state (Figure 3a,b). This was because the oxygen-containing functional group of GO broke the C=C double bond and resulted in a wrinkled state. As can be seen from Figure 3a,b, the GO layer grew on the surface of the Ag@Au NPs and GO was coated on part of the sensor. Without GO, the surface of the sensor was full of Ag@AuNPs, just like in Figure 3b. This is also illustrated by the polarizing microscope image in Figure 2a,b, where GO is only coated on a partial area of the sensor. As shown in Figure 3c,d, the morphology characterization of GO by AFM showed that GO was a two-dimensional structure with a flat surface and an average thickness of 2–4 nm, with an obvious bilayer structure.

### 3.2. Ag@Au Sensor GO/SPA Modification and SPR Characterization Tests

Dopamine is a small molecule containing both amine and catechol groups that can self-polymerize into polydopamine (PDA) films in a weakly alkaline aqueous solution. PDA has excellent biocompatibility and can be deposited on the surface of a variety of inorganic or organic substances. Due to the properties of PDA, we can use dopamine as a “double-sided tape” between the sensor and the biometric molecule, playing a good fixing role. We all know that dopamine can self-polymerize on the surface of almost any material to generate an adhesive PDA coating, which contains abundant functional groups, such as quinone, amino, carboxyl, catechol, and other groups, and that PDA is bound together by non-covalent bonding forces such as hydrogen bonding, charge transfer, and π stacking between monomers, and has the ability to re-react. In our research, we showed that the catechol group in dopamine can chelate and reduce Au^3+^ to a gold sulfite complex, which further promotes the formation of Au-Ag@Au sensors. Polydopamine acts as both a stabilizer and a binder in the reaction. Taking advantage of the universal adhesion ability and high reactivity of PDA films, GO was introduced into PDA films and then we synthesized a GO film on the surface of the optical fiber.

**Figure 4:**
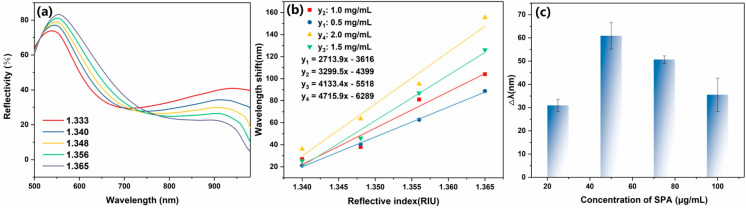
(**a**) Reflectivity spectra of the GO-coated SPR sensor in solvents with different RI values. (**b**) Comparison of refractive index sensitivity of modified GO fiber SPR sensors with different concentrations. (**c**) Modified sensor wavelength offset with different SPA concentrations.

The sensor’s reflectance spectra were measured in varying refractive indices to estimate the SPR sensor’s sensitivity after GO modification. Refractive index sensitivity is the ratio of ΔSPR to Δ*n*, where ΔSPR is the shift in the corresponding resonance wavelength and Δ*n* is the change in the solution’s refractive index. The Ag@Au/GO sensors were obtained by using PDA adhesion to modify GO to the sensor surface, and we then immersed them in different concentrations of sucrose solutions to test their reflectance spectral changes. As can be seen from Figure 4a, the resonance wave shifted with the increase in the refractive index of the solution. Compared with the same sensor without GO modification, the sensitivity of the sensor after GO modification was higher, and the sensitivity of the sensor gradually increased with the increase in GO modification concentration. As shown in Figure 4b, the sensor sensitivity was shown by the sensor immersed in different concentrations of GO, the sensor sensitivity greatly improved with the increase in GO concentration, and the sensor sensitivity reached the highest value when the GO concentration increased to 2 mg/mL, which is 4715.9 nm/RIU. Therefore, the optimal concentration of GO is 2 mg/mL. GO is a water-dispersible graphene derivative, usually obtained by oxidizing graphite in a mixture of strong acids and oxidants, and it has both a hydrophobic moiety of the original graphite structure and a hydrophilic moiety that produces oxygen-containing functional groups such as hydroxyl, epoxy, carbonyl, and carboxyl groups through an oxidation process. GO has a high dielectric constant. When modified on the gold film surface of an SPR sensor, it alters the distribution of the local electromagnetic field, enhancing the excitation efficiency of surface plasmons. Additionally, GO’s atomically thin layered structure forms close contact with the metal surface, strengthening the coupling effect of localized surface plasmons and increasing the electric field intensity [28].

We believe that the high sensitivity is due to the silver-core gold-shell structure of the sensor, and the bimetallic layer meeting the high-strength graphene oxide, which enhances the SPR signal. The antibody is efficiently immobilized on the surface of the sensor, providing more binding sites for immunoassay experiments, which is essential for antigen detection. The covalent amine conjugation of antibodies on chemically activated surfaces is a common method for antibody immobilization. However, this method’s random orientation of the antibody results in a low density of antigen binding sites and a reduction in the sensitivity of immunoassays. Staphylococcal protein A is commonly used in immunoassays for the immobilization of antibodies. SPA is a polypeptide produced by Staphylococcus aureus, which specifically binds to the fragment crystallizable (Fc) region of the antibody. Therefore, it is possible to achieve a high degree of orientation of the capture antibody on the sensor surface by SPA, but it is imperative that SPA modification concentrations be explored. The adsorption of SPA to the sensor can increase the amount of antibody fixation, which has been studied in some studies [29]. However, in the process of this experiment, it was found that the modified concentration of SPA had a greater impact on the SPR signal. Therefore, the effect of SPA concentration change on the SPR signal was explored. The SPA was modified to the Ag@Au/GO fiber SPR sensor, and the optimal modified concentration of SPA was 50 μg/mL, as shown in Figure 4c.

### 3.3. Ag@Au/GO/SPA Sensor Antibody Fixation and Antigen Detection

**Figure 5:**
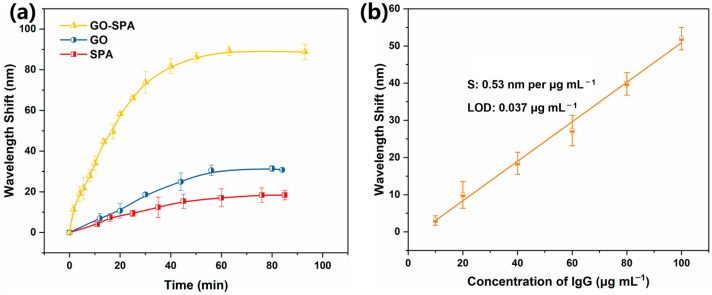
(**a**) Kinetic absorption curves of immobilized rabbit anti-human IgG at the concentration of 100 μg mL^−1^ on the GO-SPA-coated (yellow line), GO-coated (blue line), and SPA-coated (red line) SPR sensor. (**b**) Calibration curves for human IgG detection based on the GO-SPA functionalized biosensors.

Antibodies are efficiently immobilized on the surface of the sensor, providing more binding sites for immunoassays, which is essential for antigen detection. As shown in Figure 5a, the sensor was modified with GO, SPA, and GO-SPA, respectively, and immersed in 100 μg/mL rabbit anti-human IgG to bind antibody molecules. It can be seen that the amounts of fixed antibodies in the bimetallic sensor co-modified by GO-SPA were the highest. The amount of immobilized antibody obtained by the co-modification of GO and SPA to the surface of the sensor was 3 times that of the modification alone. We found that GO contained a large number of functional groups that can provide binding sites for a variety of proteins, which greatly improved the amount of antibody fixation; on the other hand, SPA can achieve a high degree of orientation to capture antibodies. The functional groups present in graphene oxide mainly include epoxy groups, hydroxyl groups, carboxyl groups, and carbonyl groups, among which there are epoxy groups and hydroxyl groups located in the structural plane, and carboxyl groups, carbonyl groups, phenol, and other groups located at the edge of the structure. As a functional group that can provide binding sites for a variety of proteins, carboxyl groups have many applications in biological detection. The functional groups rich in GO allow the antibody molecules to be immobilized on the SPR sensor with a high density of silver-nucleus gold-shell particles; the modification of SPA enables the sensor surface to achieve a high orientation of the capture antibody, and the two work together to greatly improve the amount of antibody fixation.

The standard curve of human IgG detection was obtained by immersing the Ag@Au/GO sensor immobilized with rabbit anti-human IgG antibody into different concentrations of human IgG solution (Figure 5b). The detection sensitivity of the sensor was evaluated by monitoring the resonance wavelength shift caused by the specific binding reaction of the antigen to the antibody. Figure 5b shows the standard curve of the Ag@Au/GO fiber SPR sensor for detecting human IgG. The sensitivity of the sensor for detecting human IgG was 0.53 nm/μg/mL, and the detection limit was 0.037 μg/mL. The sensitivity of the sensor to antigen detection was mainly attributed to the fact that more antibody molecules were immobilized on its surface, and the more antibody fixation, the higher the antigen detection. Table 1 shows the performance comparison table of different types of sensors, from which it can be seen that the fiber optic SPR sensor prepared by Ag@Au bimetal and GO enhancement in this study had a sensitivity of 0.53 nm/μg/mL, its human IgG detection sensitivity was higher than that of other applications, and our study presented good sensing performance.

### 3.4. Sensor Detection Specificity, Selectivity, and Stability

**Figure 6:**
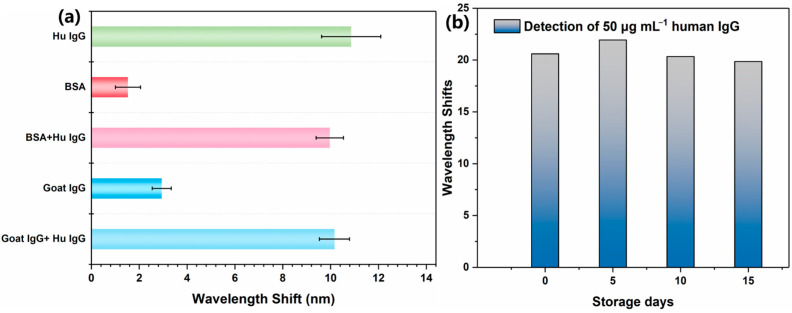
(**a**) Shifts in the resonant wavelength measured with different antigens (human IgG, BSA, BSA + human IgG, goat IgG, and goat IgG + human IgG) based on the GO-SPA biosensors. (**b**) Stability of the rabbit anti-human IgG immobilized GO-SPA SPR sensor.

To evaluate the sensor detection specificity and selectivity of the Ag@Au/GO fiber optic SPR sensor, we tested the adsorption of the reference protein (BSA and goat IgG) through the sensor immobilized with rabbit anti-human IgG antibody, and the detection of the target (human IgG) through the sensor in a multi-component solution (BSA + human IgG and goat IgG + human IgG). From Figure 6a, we can see that the reference proteins caused a slight wavelength shift (1.73 nm for BSA, 3.16 nm for goat IgG) and the wavelength shifts in target human IgG in multicomponent solutions (10.08 nm for BSA + human IgG and 10.23 nm for goat IgG + human IgG) were close to that (11.12 nm) of human IgG alone. These results suggest that the sensor exhibited high selectivity. In order to determine the detection stability of the sensor, the sensor with fixed antibodies was stored in a −4 °C freezer for 0 days, 5 days, 10 days, and 15 days, respectively. Then, the sensing area was immersed in 50 μg/mL human IgG solution to determine its wavelength offset (Figure 6b). As shown in Figure 6b, it showed that the sensor detection signal still remained stable after a long period of cryogenic storage.

## 4. Conclusions

We developed an innovative method to detect human IgG using a bimetallic and graphene oxide-reinforced fiber optic SPR sensor. The sensor is characterized by high efficiency, high sensitivity, and low detection limit. With the increase in GO concentration, the RI sensitivity was significantly improved, the optimal concentration was finally determined to be 2 mg/mL, and the highest RI sensitivity of the Ag@Au/GO fiber SPR sensor was 4715.9 nm/RIU in the range of 1.333–1.365. The sensitivity of the human IgG antigen test was 0.53 nm/μg/mL and the detection limit was 0.037 μg/mL. Furthermore, the proposed SPR sensor also offers excellent specificity and stability.

## Figures and Tables

**Table 1:** Comparison of the performance of various types of sensors.

Analyte	Modified Materials for Sensors	RI Sensitivity (nm/RIU)	Analyte Detection Sensitivity (nm/μg/mL)	LOD (μg/mL)	Ref.
Human IgG	GO/AgNPs	3311.47	0.4985	0.04	[11]
Human IgG	Ti_3_C_2_T_x_ MXene/AuNPs	2804.5	1.7046	0.17	[12]
Human IgG	Carbon nanotubes	5948.57	3.272	0.006	[24]
Human IgG	GO/SPA	4649.8	/	0.01	[30]
Analyte	Double-layer Au NPs/GO	3436.2	/	/	[25]
Analyte	Au-Ag@AuNPs	3512	/	/	[23]
Human IgG	AuNPs	2054 (1.333–1.359) 3980 (1.359–1.386)	0.41	0.9	[20]
Human IgG	Ag@AuNPs/GO	4715.9	0.53	0.037	This work

## Data Availability

The data are available on reasonable request from the corresponding author.
